# Mitochondrial *Cox1* Sequence Data Reliably Uncover Patterns of Insect Diversity But Suffer from High Lineage-Idiosyncratic Error Rates

**DOI:** 10.1371/journal.pone.0014448

**Published:** 2010-12-28

**Authors:** Lars Hendrich, Joan Pons, Ignacio Ribera, Michael Balke

**Affiliations:** 1 Department of Entomology, Zoological State Collection, Munich, Germany; 2 GeoBioCenter, Ludwig-Maximilians-University, Munich, Germany; 3 Departament de Biodiversitat i Conservació, Institut Mediterrani d'Estudis Avançats, Esporles, Spain; 4 Institute of Evolutionary Biology, Barcelona, Spain; Institute of Evolutionary Biology (CSIC-UPF), Spain

## Abstract

**Background:**

The demand for scientific biodiversity data is increasing, but taxonomic expertise is often limited or not available. DNA sequencing is a potential remedy to overcome this *taxonomic impediment*. Mitochondrial DNA is most commonly used, e.g., for species identification (“DNA barcoding”). Here, we present the first study in arthropods based on a near-complete species sampling of a family-level taxon from the entire Australian region. We aimed to assess how reliably mtDNA data can capture species diversity when many sister species pairs are included. Then, we contrasted phylogenetic subsampling with the hitherto more commonly applied geographical subsampling, where sister species are not necessarily captured.

**Methodology/Principal Findings:**

We sequenced 800 bp *cox1* for 1,439 individuals including 260 Australian species (78% species coverage). We used clustering with thresholds of 1 to 10% and general mixed Yule Coalescent (GMYC) analysis for the estimation of species richness. The performance metrics used were *taxonomic accuracy* and *agreement* between the morphological and molecular species richness estimation. Clustering (at the 3% level) and GMYC reliably estimated species diversity for single or multiple geographic regions, with an error for larger clades of lower than 10%, thus outperforming parataxonomy. However, the rates of error were higher for some individual genera, with values of up to 45% when very recent species formed nonmonophyletic clusters. *Taxonomic accuracy* was always lower, with error rates above 20% and a larger variation at the genus level (0 to 70%). Sørensen similarity indices calculated for morphospecies, 3% clusters and GMYC entities for different pairs of localities was consistent among methods and showed expected decrease over distance.

**Conclusion/Significance:**

*Cox1* sequence data are a powerful tool for large-scale species richness estimation, with a great potential for use in ecology and β-diversity studies and for setting conservation priorities. However, error rates can be high in individual lineages.

## Introduction

The overwhelming number of described and undescribed species as well as the alarming loss of taxonomic expertise globally [1] raise the question of how to expedite taxonomic identification [2]. DNA sequence data has been advocated as a potential remedy for this taxonomy crisis (for example, DNA taxonomy: [3], [4]; DNA barcoding: [5], [6]). For barcoding, the sequencing of 648 base pairs of the 5′ end of the mitochondrial cytochrome c oxidase 1 (*cox1*) gene has become the most widely used approach (www.barcodinglife.org). Proponents of this method remain enthusiastic [7], and criticism of it, which was passionate initially, is now more focused on its pitfalls (e.g., widespread introgression or incomplete lineage sorting: [8]; nuclear copies of mitochondrial genes: [9]). DeSalle [10], [11] clarified many misconceptions related to the use of *cox1* sequence data as a means of species identification, highlighting the enormous potential of *cox1* sequences to generally *diagnose* species reliably, although never neglecting other diagnostic data sources, such as morphology.

Animal DNA barcoding is mainly focused on the mitochondrial *cox1* gene because mitochondrial DNA is highly abundant in the cell; its amplification is comparably reliable; and *cox1* is often variable from populations to higher taxonomic levels [5]. An underlying assumption for species recognition through DNA barcoding and, indeed, for any other DNA sequence-based approach is that intraspecific sequences are more similar to each other than to sequences from other species. Ideally, within-species divergence should be very small, while divergence from sister species and all other species is larger. Using data for congeneric species from GenBank, Hebert *et al.* [6] suggested that such a *barcoding gap* does in fact exist, which was further exemplified by a study of 260 species of North American birds [12] and other studies in which interspecific variation was found to be 10 times higher on average than intraspecific divergence. While this might often be the case, several studies have countered that increased sampling does not simply increase the accuracy of molecular identification. Rather, denser sampling, or sampling of lineages rather than geographical areas, may decrease accuracy as it increases the probability of including sister species or very closely related species that might not be present in a sub-sampling of the lineage. In such cases, intra- and interspecific sequence divergences can be zero, small and/or broadly overlapping [13], [14], [15], [16], [17]. Increasing sampling density can be seen as a shift from simple regional subsampling towards clade-biased subsampling.

In extreme cases, mtDNA variation might appear to be taxonomically unstructured due to incomplete lineage sorting and/or an imperfect existing taxonomy, which inevitably causes morphospecies to form para- or polyphyletic clusters [8], [18], [19]. Kerr *et al.* [20] argued that such shortcomings might mainly occur in what they called “extreme situations” and taxonomically poorly studied groups. Tropical islands, such as Fiji, are often thought to represent such “extreme situations”, where recent bursts of diversification with possibly related mtDNA idiosyncrasies have intuitively been postulated. While this might be the case [8], other studies have argued against making generalizations [21] and concluded that it is not possible to predict where mtDNA and morphological species identifications will reveal congruent or incongruent groups, even when closely related taxa are considered.

Thus, it appears that an exploration across taxonomically diverse assemblages and over large areas and different biomes maybe desirable to compare the performance of geographical vs. phylogenetic sampling of a lineage. However, studies conducting comprehensive species-level sampling of a larger monophyletic group or of higher taxa across a biogeographic region remain surprisingly scarce, despite the fact that DNA-based taxonomies can suffer from large error rates due to incomplete sampling of species or populations [22]. Regional datasets might underestimate intraspecific variation or, more seriously, undersample closely related species and, thus, overestimate interspecific divergences [13]. In one of the first comprehensive studies of this type, Meyer & Paulay [13] analyzed 263 taxa, representing more than 93% of the recognized world species of cowries (Mollusca: Cypraeidae). Of 218 traditional cowrie species tested, 18 (8%) were polyphyletic with respect to another recognized species, presumably their sister. Meyer & Paulay [13] suggested these were either young species suffering from incomplete lineage sorting or artificially split forms for which the current taxonomy blurred species boundaries. Dense taxonomic sampling capturing sister species pairs is, therefore, a prerequisite for testing the possibilities and limitations of sequence-based methods. Monaghan et al. [23] conducted regional sampling of insects from four orders and 12 families from five sites in Madagascar, with an estimated taxonomic coverage of Madagascan γ-diversity of 20% to 80% between the different taxa. Employing the general mixed Yule coalescent method (GMYC), 370 putative species were recognized that were in general agreement with morphologically delineated entities (up to 6% overestimation of species number).

Here, we explore how well the clustering of mtDNA sequences captures the species diversity of diving beetles (Dytiscidae) across the entire Australian continent. Australia is biogeographically fairly isolated from the rest of the world and includes extensive and taxonomically well-known monophyletic radiations of Dytiscidae [24]. This diversity, coupled with dramatic past and present climate change [25], a mixture of tropical, temperate, lowland and highland biota, and manageable logistics suggested Australia as a study area. This ecologically and phylogenetically diverse setting should contain older as well as potentially rather recently diversified clades. Australia has the conditions to qualify as both a museum and cradle of biological diversity.

We used a comprehensively sampled group in an ecologically diverse region to answer the following questions: 1) to what extent do *cox1* data corroborate morphospecies hypotheses (taxonomic accuracy of clustering); 2) is the number of *cox1* groups an acceptable proxy delimiting species diversity (agreement between cluster or GMYC entity number and morphospecies count); 3) can *cox1* data help to uncover patterns of species diversity; and 4) what, if any, are the effects of analyzing regional subsamples instead of whole lineages?

## Materials and Methods

There is high demand for rapidly releasable, quantitative biodiversity data. Fast clustering analyses are widely implemented using barcoding technology, but the underlying fixed-threshold approaches have rightly been criticized as biologically meaningless [23] or as phenetic [11]. The incorporation of DNA sequence data in the aim of identifying and formally describing all species is a task for integrative taxonomic studies carried out by taxonomic researchers using multiple data sources to formulate sound species hypotheses [11]. Here our aim was to ask how well mtDNA sequences capture species diversity in larger samples, and for this, we used a threshold-based, fast clustering approach. For comparative purposes, we also employed the general mixed Yule coalescent method (GMYC) [26] to delimit groups of haplotypes corresponding to intraspecific genetic diversity.

### Sampling and taxonomy

Diving beetles have a worldwide distribution, with all main phylogenetic lineages present in several major biogeographical regions [27]. The Australian fauna is a composite of several lineages, some of which are represented by species in widely distributed genera (>150 spp.). The rest (>180 spp.) are distributed among 20 endemic genera belonging to different subfamilies and tribes [17], [24]. The otherwise mainly Holarctic tribe Hydroporini has diversified extensively in Australia, forming a radiation of 10 morphologically and ecologically very diverse genera and >150 described species. Similarly, there are several diverse clades within the tribe Bidessini, including *Limbodessus* (with an extensive radiation in the subterranean waters of western Australia) and *Neobidessodes* and, finally, approximately 30 species in the Copelatinae genus *Exocelina.*

The goal of this study was to maximize the species level sampling density for the diving beetles of the Australian continent, particularly for the multiple endemic Australian radiations. We collected more than 10,000 specimens from approximately 200 localities. The samples were sorted into morphospecies by one of us (LH) who has extensive experience with these Australian fauna, performing several sorting iterations to maximize the number of species identified for this relatively large number of ethanol-preserved specimens. When available, up to 50 males were dissected for the examination of genital structures relevant to identification. Then several (preferably male) individuals per morphospecies were selected for DNA extraction (average 4.8, up to 32 in the widespread species *Rhantus suturalis*). These specimens were ideally from as many localities that were as widely distributed as possible. Twenty-six species were represented by singletons. Most specimens were identified to the species level, or they were assigned to morphospecies when taxa require taxonomic revision (e.g., genus *Exocelina*).

We sequenced 1,141 epigean Australian specimens; we also sequenced 112 specimens from adjacent geographical areas (New Guinea, Fiji, New Zealand and New Caledonia) to cover species with wider geographical ranges than continental Australia and to include the few species of otherwise strictly Australian genera that are endemic to neighboring islands.

The Australian diving beetle fauna is outstanding because it features a very diverse underground (stygobiont) fauna, with 99 species described to date [28], most of which are in the Hydroporinae: Bidessini (*Limbodessus*) and Hydroporini (*Paroster*). We could not sample these habitats, but 65 sequences for 61 stygobiont species were downloaded from GenBank. Species of *Hygrobia* (Hygrobiidae), a family closely related to the Dytiscidae [27], were used as an outgroup.

After the analysis of *cox1* sequences, every specimen was again inspected by a taxonomist to correct possible misidentifications and, in many cases, to improve the existing taxonomy by a more detailed comparison of male genitalia and other structures. For some groups, we prepared taxonomic revisions based on thousands of dried specimens, often dissecting morphological structures for dozens of individuals, e.g., [17].

We compiled different datasets for the analyses: 1) all data combined, 2) phylogenetic subsampling, with datasets containing all of the available species from different endemic Australian radiations (to test the effect of dense taxon sampling), and 3) regional datasets that each contained all of the specimens from a given area (individual or combinations of Australian states).

### Sequencing

DNA was extracted using the DNeasy Animal Tissue kit (Qiagen, Hilden, Germany). We sequenced the 3′ end of *cox1* using the primers Jerry (F: CAA CAT TTA TTT TGA TTT TTT GG) and Pat (R: TCC AAT GCA CTA ATC TGC CAT ATT A) [29]. Although this is not the fragment proposed as a standard barcode (5), our results can be generalized because the average evolutionary rate of both *cox1* fragments is similar. Roe & Sperling [30] sequenced and evaluated the information from the entire *cox1*–*cox2* region, showing that “ultimately, no single optimally informative [for barcoding] 600 bp location was found within the 2.3 kb of COI–COII, and the DNA barcoding region was no better than other regions downstream in COI”. Our results may also, to some extent, apply to any mitochondrial protein-coding gene with a similar evolutionary rate, as problematic issues with mitochondrial DNA related to species delineation are linked to the mitochondrial genome *per se* rather than individual genes [8], [26]. Sequences were edited in Sequencher 4.8 (Genecodes Corp., Ann Arbor, MI, USA) and translated into amino acid sequences for alignment control and screening for internal stop codons or other anomalies in MacClade [31]. Finally, nucleotides were aligned using MUSCLE [32] under default settings on CIPRES Portal v.2 (www.phylo.org). New sequences have been submitted to GenBank.

### Phylogenetic analyses

Parsimony searches were run in the program TNT version 1.1, which we also used to run 500 jackknife (character removal 36%) replications to assess node stability [33] (hit best tree five times command, keeping 10,000 in memory). We ran maximum likelihood (ML) analyses with the program GARLI [34] on CIPRES Portal v.2. We used the GTR+I+G model as selected by MrModeltest for the combined dataset and ran analyses until 10,000 generations revealed no significant improvement of the likelihood scores of the topology. Bootstrap values were based on 250 replicates using only the datasets of the different Australian radiations due to computational limitations. We also ran ML analyses in RAxML v7.0.4 [35], bipartitioning the data (1st +2nd *versus* 3rd codon sites) and implementing a GTR model with CAT approximation to incorporate rate heterogeneity across sites. Haplotype networks based on statistical parsimony [36] were calculated using TCS 1.13 ([37]; 95% connection limit). This approach subdivides the variation based on the level of homoplasy within the data themselves, i.e., distinguishes between long (homoplastic) and short (non-homoplastic) branches, which provides a relative measure of the divergence within a given dataset, rather than using *a priori* determined thresholds. Independent haplotype networks generally agree with named species or species groups [26], [38].

### Group delineation: clustering, character-based, GMYC

We ran a neighbor-joining analysis using uncorrected p-distances for fast distance-based clustering of the data. The SpeciesIdentifier module of TaxonDNA software v.1.6.2 was used to study the genetic divergences in our dataset and to cluster sequences at different preset thresholds using uncorrected p-distances ([14]; http://code.google.com/p/taxondna/). SpeciesIdentifier accounts for threshold violations according to triangle inequity (i.e., when the divergence between A – B and B – C is 3% or less, but A – C exceeds 3%, then A, B and C would still be grouped into one 3% cluster by Taxon DNA). SpeciesIdentifier recognizes *a priori* delineated species from the sequence name, as long as the name follows the format “Genus species”, i.e., “*Rhantus suturalis*”, or “*Rhantus* australiaone MB1307”. The output summarizes the number of different species names in the dataset, the number of clusters found under the present threshold (e.g., 1%, 2%), the number of clusters that contain only one species name, and the number of *perfect clusters* (those that contain all individuals under one species name and only those individuals, i.e., monophyly). Thus, we can calculate the number of split clusters (one species split into more than one cluster, i.e., paraphyly) and lumped clusters (more than one species name in a cluster). SpeciesIdentifier was used for species richness estimation, with clusters taken as species surrogates. For any clustering threshold (e.g., at 1%, 2%, 3%…), two values were reported. The first of these values was the number of clusters found relative to the number of morphology-based species names in the dataset (*agreement* hereafter). For example, a dataset with a hundred species names and a threshold clustering at 25% divergence would likely reveal only one cluster. Thus, our species richness estimation would amount to a meager 1% (*agreement*) of the actually present species as delineated by morphology. Second, and more importantly, we report *taxonomic accuracy*, which was calculated as the number of perfect clusters (i.e., clusters containing all sequences of a morphology based species and only those sequences) relative to the number of species in the dataset. The number of perfect clusters can increase when the existing taxonomy is revised to accommodate cryptic or overlooked species. A one hundred percent *accuracy* means that all clusters perfectly mirror the species hypotheses based on morphology.

Character-based group delineation, or population aggregation analysis (PAA) [39], was used to delineate geographically endemic subgroups or species within groups *a priori* identified by clustering and phylogenetic analyses. The sequences of the species were manually screened for diagnostic characters in the DNA sequence editor Se-Al (http://tree.bio.ed.ac.uk/software/seal/). Specifically, we applied PAA to several supposedly recent morphospecies that were lumped into paraphyletic species clusters. In a pairwise step addition, PAA defines populations based on the presence of at least one diagnostic (fixed) character in one population; otherwise, they are merged and then compared to another population. For species delineation, taxonomists traditionally use diagnostic characters from morphology or behavior, and this usage can be extended to nucleotide characters [11], [40].

We also implemented the method developed by Pons *et al.* [26] and explained in detail in a previous publication [23] that delineates genetic clusters using a generalized mixed Yule coalescent (GMYC) model that represents independently evolving entities. This method uses a maximum likelihood approach to optimize the shift in the branching patterns of the gene tree from interspecific branches (Yule model) to intraspecific branches (neutral coalescent). The model optimizes the maximum likelihood value of a threshold, such that the nodes before the threshold are identified as species diversification events, while the branches beyond the threshold are clusters following coalescent processes. This method has previously been implemented in other taxonomically understudied groups [23], [26], [41], [42], [43]. In large trees, a unique species-populations split for a particular time (single threshold) may not reflect the true diversification for all of the lineages included; therefore, we performed an analysis allowing multiple and independent thresholds over time and across the tree [23]. Generalized mixed Yule coalescent clustering was performed here using the R package SPLITS (SPecies' LImits by Threshold Statistics), which allows single or multiple thresholds (http://r-forge.r-project.org/projects/splits/). This package provides confidence intervals (CI) in the output (solutions within two log-likelihood units of the maximum likelihood), but the GMYC entity content of these solutions cannot be retrieved in the output at present. Before running SPLITS, an ultrametric tree was made fully dichotomous, and branches with zero branch length were pruned or removed using the package ape in R [44]. The underlying tree was derived from our above RAxML v7.0.4 analysis. Identical sequences were removed from the analysis using the reduced dataset provided by RAxML. ML tree searches were run 100 times starting from different parsimony trees, and the best one tree was finally optimized. Branch lengths were made ultrametric using PATHd8 software [45] by arbitrarily setting the root node to 100 Ma. This age was chosen because it approximately renders the so called standard rate of nucleotide substitution of 2.3% per Ma in insects [46]. The standard rate, which was suggested for the species and genus levels based on several arthropod examples, roughly agrees with the rates reported for different groups of Coleoptera based on calibrations using different biogeographical events [47], [48], and for the whole Coleoptera using fossils [49].We report the number of GMYC entities and the number of perfect GMYC entities containing all and only the members of an *a priori* identified species.

### Diversity patterns

The Sørensen similarity index [50] was calculated to compare samples from two sampling regions, which were states in this analysis. The index is S = 2C/A+B, where A and B were the number of species, clusters or GMYC entities in samples A and B, and C is the number of species, clusters or GMYC entities shared by the two samples. The distance between sampling regions was measured between the approximate center of all localites for each state in GoogleEarth. The distances between localities as used here are given in Table 1.

**Table 1:** *Agreement* and *taxonomic accuracy* in regional and whole-fauna clustering at 3%.

clustering at 3% region	sequenced species number	cluster number	number of shared clusters	shared cluster %	species richness estimation success %	number of perfect clusters	taxonomic accuracy %	distance between localities
**TAS**	16	12			85	11	69	0
**NT**	73	69			94	64	88	0
**VIC**	32	30			94	27	84	0
**WA**	76	78			102	69	91	0
**QLD**	73	70			96	57	78	0
**NSW**	59	56			95	44	75	0
**SA**	25	24			96	18	72	0
**mean (SD) median**					**94 (4.6) 95**		**79 (7.6) 78**	
**TAS-NT**	89	81	0	0	91	75	84	3200
**NT-VIC**	103	98	1	1	95	88	85	2700
**QLD-WA**	140	138	10	7	99	113	81	2700
**NSW-WA**	129	128	6	5	99	105	81	2300
**TAS-WA**	92	89	1	1	97	78	85	2100
**NT-WA**	141	138	9	7	98	123	87	2100
**TAS-SA**	36	30	6	20	83	24	67	2000
**QLD-TAS**	86	80	2	3	93	64	74	1500
**QLD-SA**	92	88	6	7	96	69	75	1400
**NT-QLD**	115	111	28	25	97	90	78	1300
**TAS-NSW**	66	60	8	13	91	46	70	500
**TAS-VIC**	40	34	8	24	85	29	73	250
**QLD-NSW**	111	100	26	26	90	76	68	10
**VIC-NSW**	67	60	26	43	90	48	72	10
**mean (SD) median**					**93 (4.9) 94**		**77 (6.5) 76**	
**QLD-NSW-VIC**	117	104			89	80	68	
**QLD-NSW-VIC-TAS-NT**	164	148			90	114	70	
**All species**	315	288			**90**	230	**73**	

Abbreviations: TAS  =  Tasmania, NT  =  Northern Territory, VIC  =  Victoria, wa  =  Western Australia, QLD  =  Queensland, NSW  =  New South Wales, SA  =  South Australia.

## Results and Discussion

### Sequencing and phylogenetic analyses

**Figure 1:**
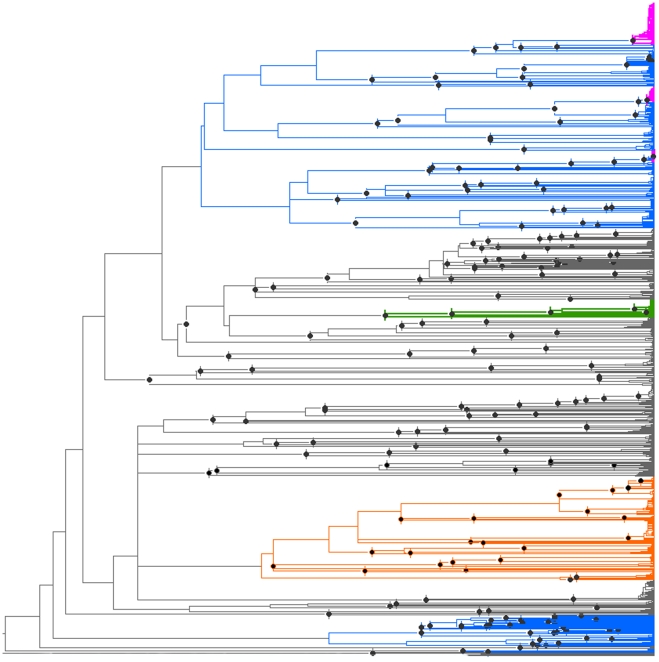
Chronogram of cox1 sequences for the Australian diving beetle fauna based on ML branch lengths which were made ultrametric with a relaxed molecular clock. Focal clades are Hydroporini (blue, polyphyletic); *Neobidessodes* (green) and *Exocelina* (orange). Three pink clades contain paraphyletic species. Dots denote speciation events as inferred from morphospecies identification or combined genetic and morphological data. Note: Multiple dots within the pink clades omitted for clarity.

The 828 bp alignment was free of indels. Amino acid translation neither showed stop codons nor aberrant non-synonymous amino acid substitutions. The final dataset of 1,439 *cox1* sequences included 1,141 sequences of 260 morphologically recognized Australian species, representing 78% of the 331 described Australian Dytiscidae species (as of November 2009). We covered 199 species (86%) of the 232 described epigean species and 61 (60%) of the 99 stygobiont species. From the radiations of the Australian endemic Hydroporini genera, we sequenced 109 of 130 known epigean species (83%), including the few members occurring in Fiji, New Caledonia, New Zealand and New Guinea (e.g., *Megaporus tristis* from Fiji and *Chostonectes maai* from Papua New Guinea). After adding stygobiont species data from GenBank, we had a total of 125 Hydroporini species (83% of the total 150 Australian Hydroporini). Most higher taxa, such as genera and tribes, were recovered as monophyletic (Figure 1). Exceptions were the genus *Carabhydrus* (three included species) and the genus *Paroster* (45 species), which grouped outside the remainder of the Hydroporini clade (Figure 1, outsiders: lower blue portion). Most of the species were retrieved with >50% parsimony jackknife and maximum likelihood bootstrap support from GARLI (Table S1).

Thirty misidentified specimens/species were obvious from the neighbour joining (NJ) and parsimony trees, as well as the SpeciesIdentifier clusters, which were run for data quality control. Misidentifications also included taxonomic misconceptions, such as the widespread, large, conspicuous species *Exocelina australasiae* and *E. melanarius*, which formed multiple paraphyletic clusters. Morphological reinspection revealed different shapes of male claws and copulatory structures, which were indicative of 6 instead of two species. This motivated us to carefully inspect all of the specimens in the dataset again for possible taxonomic misidentifications.

Our dataset included 34 new Australian species discovered through an iterative process of morphospecies sorting, sequencing, morphological reexamination of separate clusters and taxonomic revision [17], [51]. Most of these new species were specimens that we failed to assign to a known species, which were assigned operational names such as “*Exocelina* smallbrown”. Other possibly new species were cryptic and misidentified as known species. One *Antiporus* cluster (initially identified as *A. femoralis*) and one *Sternopriscus* cluster (identified as *S. clavatus*) diverged genetically from what were thought to be conspecific specimens by 6.9 to 7.2% (mean 6.4%) and 3.8 to 4.7% (mean 3.7%), respectively, though they did not diverge at all morphologically. Further investigation using ecological niche modeling and nDNA sequencing suggested that *A*. *femoralis* consists of two allopatric species [Hawlischek et al. in prep.], while *Sternopriscus* is still under study. Among the very small *Uvarus* species (body length <2 mm) were three clades diverging from each other by more than 10%. Members of these clades will be described as two new species. The five *Hydroglyphus basalis* individuals examined formed two clusters that were more than 8% divergent, one of which represents a new species. The new species of *Uvarus* und *Hydroglyphus* could also be well characterized by their male genital structures, but the results of the analyses of the sequence data were the trigger for improving the existing taxonomy. One female out of 17 *Megaporus hamatus* individuals diverged from all others by 5.5% in the absence of any morphological difference. This divergence is relatively high [6], and we are currently attempting to amplify additional markers to better understand this case.

Statistical parsimony analysis as implemented in the program TCS was applied to the Australian radiations for which we had relatively dense taxon sampling. The success of the species richness estimations was high (median 98%, mean 89%, SD 15.8%), although the taxonomic accuracy was slightly lower (median 93%, mean 83%, SD 22.6%) (Table 1).

### Cluster Analysis by Genetic Distance

The largest intraspecific uncorrected *cox1* p-distances recorded by SpeciesIdentifier were small (median 1.25%, mean 1.94%, SD 2.37%), and the average intraspecific distances were even smaller (median 0.50%, mean 0.71%, SD 0.80%).

Between species, the smallest interspecific distances within each genus were larger (median 7.42%, mean 6.51%, SD 4.14%), which increased to a median of 8.58% (mean 8.15%, SD 3.07%) after removal of morphospecies forming non-monophyletic clusters (pink clusters in Figure 1, see below). In 69% of all of our sequenced specimens, the intraspecific distance was less than 2%, and the interspecific distance diverged by more than 2% in 85% of the specimens.

Using all of the data obtained, i.e., including the non-monohyletic morphospecies, the smallest congeneric interspecific distance was clearly bimodal, with a smaller frequency peak at ca. 1% divergence, fully overlapping with the largest intraspecific distance (Figure S1). When the non-monophyletic morphospecies were removed from the dataset, the distribution of the smallest congeneric interspecific distance was approximately unimodal, with a peak at approximately 10% divergence and, thus, a clearer separation from the largest intraspecific distance. However, there was overlap between the two distributions and, therefore, no clear barcoding gap could be identified, as has been documented in other studies with dense sampling [13] (Figure S1). However, in some cases where a barcoding gap was not observed, morphological species were still diagnosable at the molecular level due to fixed nucleotide substitutions, e.g., *Neobidessodes samkrisi* and *N. flavosignatus* [17] diverge by as little as 0.85 to 1.14% but are diagnosable by five fixed nucleotide characters.

**Figure 2:**
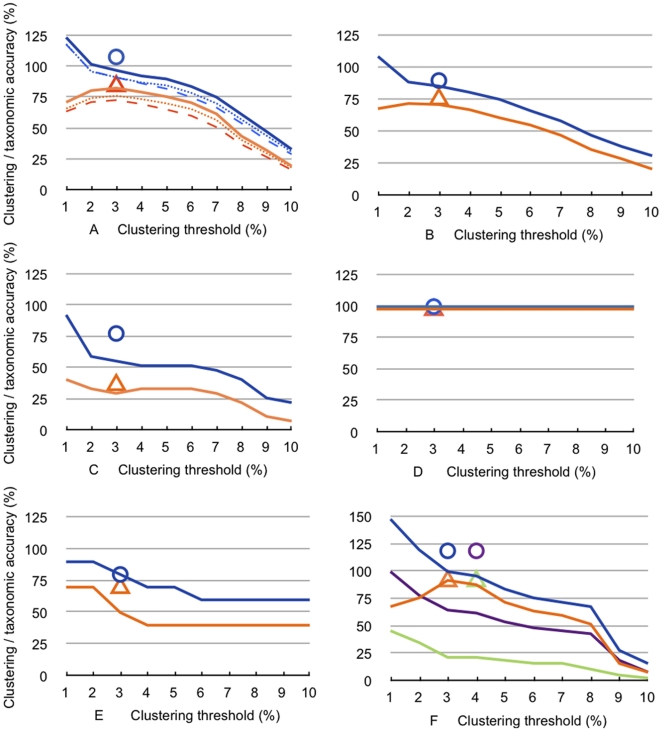
*Agreement* and *taxonomic accuracy* of molecular clusters estimated with different thresholds of DNA sequence divergence and the GMYC algorithm. Blue lines, *agreement* derived from clustering **(percentage of clusters relative to number of morphological species used in the particular dataset)**, and orange lines, *taxonomic accuracy* of clusters **(number of clusters containing all sequences of a named species and only those)**. (A) Full dataset and two modifications thereof (315 species: dashed lines; 260 species: dotted; 242 species: solid lines); (B) all Hydroporini; (C) Hydroporini: *Sternopriscus*; (D) Hydroporini: *Tiporus*; (E) Hydroporini: *Megaporus*; (F) Copelatinae: *Exocelina*, purple and green – agreement and taxonomic accuracy for the raw dataset, blue and orange – taxonomically revised dataset. Circles - *agreement* for GMYC entities, triangles – *taxonomic accuracy* of GMYC entities (in F, purple and green GMYC for taxonomically raw *Exocelina* dataset).

Using the complete dataset, with 1,439 individuals and 315 described and undescribed species from Australia and neighboring areas, the number of clusters fully agrees with the number of *a priori* identified species at a sequence similarity threshold between 1 and 2% (Figure 2A). Below that point, the number of clusters was larger than the number of recognized morphospecies (oversplitting), and above that point, the number was lower (lumping of morphospecies). Taxonomic accuracy was approximately 80%, and the highest accuracy was always achieved at a clustering threshold of 3%.

The removal of all ambiguously identified non-Australian individuals as well as most Australian *Platynectes*, which we currently cannot reliably sort to morphospecies (leaving 1,141 sequences of 260 identified Australian species), had little effect on the clustering accuracy (72.6%, Figure 2A). Finally, from these 260 species, we removed 18 species that always formed para- or polyphyletic clusters (see below). This dataset contained 242 species and 997 sequences (Figure 2A) and revealed the best taxonomic accuracy, with 82.7% of clusters actually representing species as delineated by the taxonomist.

**Figure 3:**
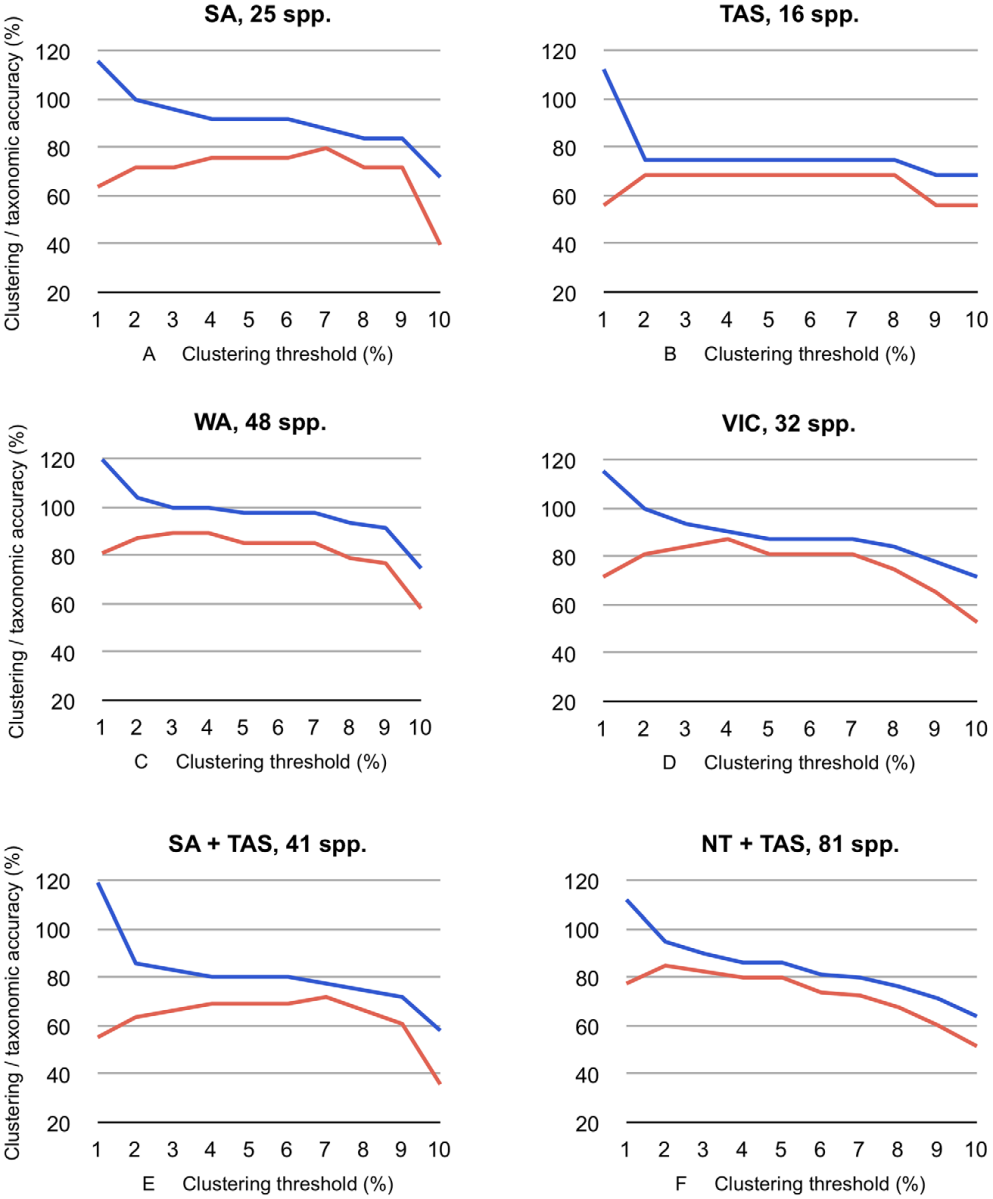
*Agreement* and *taxonomic accuracy* at different thresholds of genetic DNA distance clustering using regional subsampling (single states and pairwise comparisons), and number of species in the region(s) sampled. Blue lines: *agreement* between cluster and morphospecies number, orange lines: *taxonomic accuracy*. NT, Northern Territory, SA, South Australia, TAS, Tasmania, WA, Western Australia.

We also analyzed phylogenetic and regional subsets (Figures 2 and 3, Table 1). For the phylogenetic subsampling, we used genera and tribes. The combined 568 sequences of the 125 available species of the endemic Australian Hydroporini radiation, including species in paraphyletic clusters, revealed a similar overall trend as for the large dataset (Figure 2B), with a species richness estimation of 86% at a 3% threshold and an accuracy of 71%. However, clustering each Hydroporini genus separately showed differently structured sequence variation (e.g., Figure 2C–E), with e.g., *Tiporus* species always being perfectly clustered (Figure 2D) and *Chostonectes* exhibiting between 3 and 8% accuracy.

In the genus *Exocelina*, for which we obtained 206 sequences, we initially identified 37 morphospecies. Clustering of this dataset performed poorly in terms of both agreement and taxonomic accuracy (in Figure 2F purple and green graphs, accuracy 21% at a similarity threshold of 3%). However, after obtaining this result, a re-investigation of morphological structures and improvement of the taxonomy reduced the species count to 26, and the clustering performance of the revised dataset improved dramatically. The species richness estimation was 100% at a 3% threshold, with a clustering accuracy of 92% (Figure 2F). Thus, morphological revision opened a barcoding gap in this case. Two species (in the *E*. *australasiae* species complex) formed paraphyletic clusters, though these were resolved in separate statistical networks. Additional research will be required to arrive at sound species hypotheses in these cases.

### GMYC entities

For the GMYC analysis, we used 1082 sequences from 285 morphospecies following computationally necessary removal of identical haplotypes and zero branch lengths after clock-like transformation. The number of GMYC entities was 310 (confidence interval 307–315) using a single threshold and 405 (confidence interval 405–418) under the multiple threshold option. The single threshold option was statistically preferred over the multiple threshold (comparison of single and multiple threshold GMYC: Chi-square 4.29, d.f. 12, p = 0.98, n.s.). Among the focal groups, we found 113 GMYC entities in the Hydroporini, 8 in *Neobidessodes* and 31 in *Exocelina* (Table 2).

**Table 2:** *Agreement* and *taxonomic accuracy* using clustering at a preset threshold of 3%, single threshold GMYC analysis and statistical parsimony.

clade (species covered %)	sequenced species number	cluster number (3% clustering)	number of GMYC entities	number of parsimony networks	species richness estimation success % (3% clustering)	species richness estimation success % (GMYC)	species richness estimation success % (networks)
***Antiporus (73)*** *	11	12	12	11	109	109.1	100
***Barrethydrus (100)***	3	3	3	3	100	100	100
***Carabhydrus (30)***	3	3	3	3	100	100	100
***Chostonectes (83)***	5	5	5	5	100	100	100
***Megaporus (90)***	10	8	8	8	80	80	80
***Necterosoma (83)***	10	6	7	7	60	70	70
***Paroster (90)***	45	44	44	44	98	97.7	98
***Sekaliporus (100)***	1	1	1	1	100	100	100
***Sternopriscus (93)***	27	15	21	13	55	77.8	48
***Tiporus (83)*** *	10	10	10	10	100	100	100
**All Hydroporini**	125	107	113	105	86	90.4	84
***Neobidessodes (100)***	9	7	8	8	78	88.8	89
***Exocelina (100)*** *	26	26	31	26	100	119	96
**mean (SD) median**					**90 (16.2) 100**	**95 (12.7) 100**	**89 (15.8) 98**

	number of perfect clusters (3% clustering)	number of perfect GMYC entities	number of perfect networks	taxonomic accuracy % (3% clustering)	taxonomic accuracy % (GMYC)	taxonomic accuracy % (networks)	
***Antiporus (73)*** *	10	9	10		81.8	91	
***Barrethydrus (100)***	3	3	3	100	100	100	
***Carabhydrus (30)***	3	3	3	100	100	100	
***Chostonectes (83)***	5	5	5	100	100	100	
***Megaporus (90)***	5	7	6	50	70	60	
***Necterosoma (83)***	5	6	4	50	60	40	
***Paroster (90)***	39	41	42	87	91.1	93	
***Sekaliporus (100)***	1	1	1	100	100	100	
***Sternopriscus (93)***	8	10	11	30	37	41	
***Tiporus (83)*** *	10	10	10	100	100	100	
**All Hydroporini**	89	95	95	71	76	76	
***Neobidessodes (100)***	5	7	7	56	77.7	78	
***Exocelina (100)*** *	24	24	26	92	92.3	100	
**mean (SD) median**				**79 (23.6) 91**	**83 (18.6) 91**	**83 (22.6) 93**	

*dataset was taxonomically cleaned.

For GMYC analysis, dataset modified, identical and near-identical haplotypes removed.

For this dataset of 1082 individuals, the accuracy of the GMYC entities with respect to traditional taxonomy (83.8%, Figure 2A orange triangle) was better than that of clustering (73% at a 3% threshold). When paraphyletic species and the taxonomically poorly known *Platynectes* species were excluded, clustering had an accuracy similar to the GMYC estimation (82.7%, Figure 2A). Overall, for the raw dataset, GMYC resulted in approximatley 8% overestimation of the number of recognized morphospecies, while clustering resulted in approximately 7% underestimation (Figure 2A blue graph and circle). For the taxonomically revised dataset, clustering led to 3% underestimation and GMYC to 5% overestimation (data not shown).

When analyzed separately, the accuracies of the GMYC entities vs. clustering in the Hydroporini were 76% and 71.2%, respectively, and in *Exocelina* (with the revised morphospecies), they were 92.3% for both methods (Figure 2). The accuracy of GMYC delineation was higher in the small genus *Neobidessodes*, in which clustering lumped together four genetically similar species out of nine morphospecies, while GMYC correctly delineated 7 (55.5% vs. 77.7% accuracy; Table 2). General mixed Yule Coalescent (GMYC) generally performed slightly better than clustering at 3% (and other thresholds) for the genera of Hydroporini analyzed separately, with the exception of *Antiporus* (accuracy of 81.8% for GMYC vs. 90.9% for clustering), but the split of *A. bakewelli* into two GMYC entities may require further taxonomic investigation.

Among the oversplit morphospecies, in some cases, separate entities represented samples from geographically separated populations, such as for *Exocelina boulevardi*, where members from NSW and TAS were assigned to two entities (and *a posteriori* found to have some morphological differences), and for the samples of *Sternopriscus aquilonaris* from NSW and QLD. *Batrachomatus daemeli*, which was split into three entities, is a more complex example, with samples from localities NSW112 and VIC120 included in one entity, and one from each NSW82 and VIC120 in two additional entities (distance NSW82-NSW112 *c*. 620 km, NSW112-VIC120 *c*. 400 km). The latter case requires additional research involving the study of more samples and more populations.

In general, GMYC revealed equal or higher accuracy than clustering, and the number of GMYC entities was equal to or greater than the cluster and species numbers. For the larger datasets, GMYC tended to overestimate species numbers more than clustering, in agreement with some previous results (e.g., Madagascan insects from four orders and 12 families, for which overestimation was up to 6% [23]). For the Hydroporini, including the non-monophyletic species, both clustering and GMYC underestimated species numbers (Figure 2B). GMYC also has the advantage of being independent of preset threshold assumptions (Table 2). However, GMYC analysis is currently computationally more difficult to implement, especially because it requires a detour via branch length optimization and computation of an ultrametric tree and algorithms, which are prone to error if branch lengths are zero. Monaghan et al. [23] assessed the impact of different models of ultrametric branch length optimization on GMYC entity delineation, using five smaller datasets (<600 individuals, and generally <200 terminals). For example, a relaxed log normal with Yule prior resulted in a greater number of GMYC entities than other methods (strict or coalescent) because the Yule model was of inferior fit to the data. Nonetheless, the number and the extent of GMYC clusters was very similar. More empirical studies are needed to understand how to identify the optimal approach when datasets are very large and do computationally not allow for in-depth exploration runs.

### β Diversity

When the samples from each of the Australian states were clustered separately with a 3% threshold, the mean *agreement* between the cluster number and the number of morphospecies was 94%, and the taxonomic accuracy was 79%. For the pooled sequences of two states (e.g., South Australia + Tasmania), the mean values were 93% (*agreement*) and 77% (accuracy), respectively (Table 1).

The GMYC analysis of samples from each Australian state separately revealed a mean value for the *agreement* between the GMYC entity number and the number of morphospecies of 93% and a taxonomic accuracy of 81%. For groups from two combined regions (e.g., South Australia plus Tasmania), these mean values were 97% (*agreement*) and 77% (accuracy). The entity content varied with the extent of sampling. For example, all members of *Sternopriscus* from NSW + VIC combined versus all members of *Sternopriscus* from NSW and VIC when analyzed separately exhibited compatible GMYC entities. However, *Sternopriscus* from NT + VIC versus each of them analyzed separately showed partially incompatible entity delineation and content. When analyzing NT + VIC combined, the NT samples of *S. aquilonaris* were split into five entities, and *S. balkei*, *S. goldbergi* and *S. alligatorensis* were placed in separate entities. However, in the separate NT analyses, these species were all pooled into a single entity. Analysis of the full dataset resulted in the same entities as in the combined NT + VIC analysis. More research based on denser population-level sampling is needed to address this issue.

**Figure 4:**
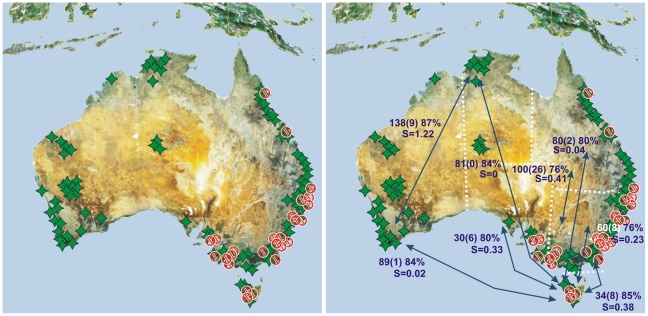
Geographical origin of samples and regional comparisons. (A) Geographical origin of sequenced Australian individuals (green stars), red  =  specimens in paraphyletic clusters. (B) Molecular biodiversity estimation employed for regional comparison. Arrows  =  states compared; Numbers  =  number of clusters using 3% threshold for all samples from the two areas compared (N of clusters shared between two areas) % of clusters that perfectly agree with existing taxonomy; S = Sørensen Index.

**Figure 5:**
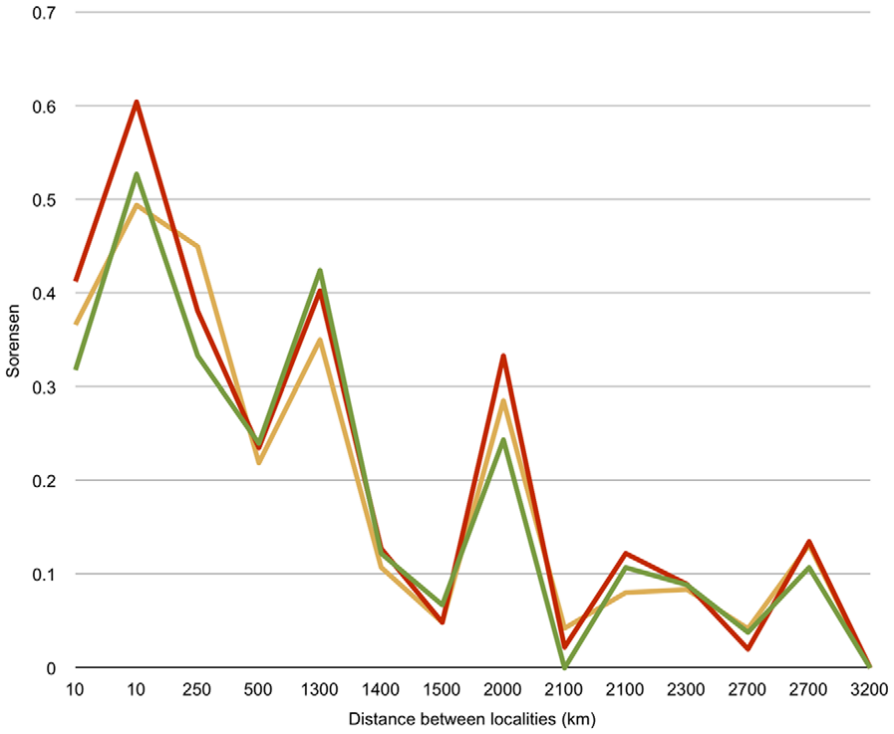
Relation between geographic distance and β-diversity (Sørensen index) for pairwise comparisons between localities. Sorensen index for species numbers based on morphology (green), numbers of *cox1* clusters, estimated at 3% threshold of genetic DNA distance (red) and derived from GMYC algorithm (orange).

Overall, the regional comparisons using morphospecies, sequence clusters and GMYC entities revealed a similar estimation of species richness and β-diversity (as measured by the Sørensen index), as well as the expected decrease in the number of shared entities over distance (Figures 4B and 5, Table 1). Krell [52] argued that phenetic morphological sorting (e.g., parataxonomy, morphospecies sorting) is highly error-prone (error rate of up to 117% species number overestimation, with a median of 22% in Krell's compilation of 79 studies), especially beyond a regional scale when dealing with many morphologically similar, vicariant species. An analysis of regional *cox1* data with an error for species richness estimation of less than 10% (Table 1) thus performs well. It is scientifically sound, as it uses repeatable concrete data and repeatable criteria, which parataxonomy does not [52]. Clustering of data from two regions revealed the same trend, with higher errors in two of the pairs (Tasmania + Victoria: 15%; Tasmania + South Australia: 17%). Clustering data from more than two regions estimated species diversity rather well (Table 1). The taxonomic accuracy depends of the species number per area, as errors are compensated by increasing sample size. Thus, we find that variance is decreased with increasing sample size. Clustering data from two areas resulted in increased taxonomic accuracy with increased distance between localities, while the degree of species richness estimation success, or the agreement between the morphospecies count and number of clusters, remained robust over distance (Table 1).

### Para- and polyphyletic clusters

Nonmonophyly between closely related species is well documented [8], [13], [18], [53] and, as expected, was a major source of error in our estimations. Incomplete lineage sorting blurring the boundary between tokogeny and phylogeny is more pronounced among recent species and may become evident when monophyletic groups are sampled thoroughly [13], as was the case in this study. Eighteen recognized species of Hydroporini were not retrieved among the monophyletic *cox1* clusters using joining-joining tree building or SpeciesIdentifier clustering. Three *Megaporus*, four *Necterosoma* and 11 *Sternopriscus* species were lumped into one cluster each. Considering a total of 260 identified Australian species, the error rate through due to species para- or polyphyly was 6.9%, even after morphological re-evaluation of non-monophyletic taxa (i.e., after the improvement of a previously imperfect taxonomy, or *reciprocal illumination* [51], [54]).

Under maximum likelihood (ML) inference, 6 additional *Sternopriscus* species were lumped into two additional clades, containing two and four species, respectively; and two additional *Necterosoma* species were also lumped. This lumping involved groups of species in which one species had no or low bootstrap support and in which individuals did not group together in the ML analysis, thus creating paraphyly relative to other species with very similar haplotypes. These species also grouped into single networks in the statistical parsimony analysis due to their haplotype similarity and were also not resolved using parsimony analysis.

In the paraphyletic *Megaporus* clade, most *M. hamatus* and *M. gardneri* specimens exhibited nearly identical haplotypes (two substitutions, 0.14% divergence) and no fixed diagnostic characters, while *M. howitti* diverged from these two species by 0.57 to 1.58%; the latter was not resolved using NJ, MP, MP analyses or clustering, but it was diagnosable by two fixed character states using population aggregation analysis (PAA). For comparison, one of 17 morphologically identical individuals of *Megaporus hamatus* (MB2239) diverged from the *M. hamatus* and *M. gardneri* specimens by >5.6%, and diagnoses through 22 fixed characters in a PAA. This might indicate the presence of a cryptic species. Four *Necterosoma* species always formed a paraphyletic cluster and only diverged 0 to 0.82%, and none of these was diagnosable. *Necterosoma darwini*, which is the next closest relative to these four species, diverged from them by only 1.26 to 1.75%, but it was readily diagnosable through one fixed nucleotide change (as a comparison, *N. souzannae*, diverging by approximately 12%, could be diagnosed through 52 characters). In the eleven para or polyphyletic species of the *Sternopriscus tarsalis* group, divergence was 0 to 3.9%, and there were no molecular characters diagnosing any of the species. The few species from the endemic radiations studied here that we did not manage to sequence (Table 1) are morphologically very distinct and do not belong to the clades containing paraphyletic species. Based on our approximate node age estimation using PATHd8, the nonmonophyletic *Sternopriscus* group diversified more recently than 2.7 MYA, while *Necterosoma* and *Megaporus* diversified more recently than 1.2 MYA. In all cases, the same paraphyletic clusters found in the NJ tree and in the clustering output appeared as paraphyletic GMYC entities.

Geographically, the members of the 18 paraphyletic species are strongly centered in mesic SE Australia (Figure 4A), and the most northern locality for species paraphyly refers to individuals of the widespread *Necterosoma undecimmaculatus*, which appears to be paraphyletic, as three other SE Australian endemic species nest within it. Massive climatic, geological and floristic transitions are well documented for this region [25]. The past five million years saw a dramatic transition of vegetation cover in the area, with forest and rainforest being replaced by more open vegetation and sclerophyllus woodlands. Climatic fluctuations between cool-dry and warm-wet conditions are documented for at least 20 glacial cycles. Finally, mountain formation, as well as marine incursions contributed to rapidly and frequently changing the abiotic environment in SE Australia [55], [56], [57]. Genetic structuring in southeastern and alpine Australian *Egernia* skinks [25] and in the common froglet *Crinia signifera* [58] was linked to these environmental fluctuations, which might well also be a motor driving the diversification of diving beetles. Our (very preliminary) nodal age estimation suggested an origin of the nonmonophyletic southeastern Australian lineages well within the periods of significant environmental transition after the Miocene – Pliocene transition (*Sternopriscus* <2.7 MYA; *Necterosoma* and *Megaporus* <1.2 MYA). Assuming alternative, faster or slower, *cox1* substitution rates than the 2% used here (1.5 to 3.5%, summarized in [47]), the nonmonophyletic groups were still determined to have originated less than five million years ago. We suggest that in this particular case, the species nonmonophyly observed is due to recent, rapid diversification in a dramatically changing landscape in which niche-building opportunities were manifold and time for lineage sorting has not yet been sufficient. Species nonmonophyly is lineage idiosyncratic, as the allopatric sister species pair *Neobidessodes samkrisi* (New Guinea) and *N. flavosignatus* (Australia) originated in the same time span as the above, but the species can be diagnosed using cox1 data [17].

### Conclusions

We employed extensive taxonomic expertise and comprehensive sampling to understand to what degree mitochondrial DNA sequences can help to obtain rapidly releasable species richness data when the data structure is supposedly diverse and the dataset contains older as well as very recent species.

We show that, for large datasets, *cox1* sequence data provide fairly precise species richness estimates using either preset thresholds (clustering) or inferences from the specific dataset itself using the GMYC approach. However, our estimates of species diversity indicate strong dependence on dataset structure. Performance at the genus level varied greatly due to idiosyncratic lineage data structures (or lineage evolution), where fixed-threshold approaches cannot accurately capture species diversity. General mixed Yule Coalescent performed better here in terms of taxonomic accuracy, but it could not overcome problems associated with species para- or polyphyly.

In this study, a small percentage of species as delineated by taxonomists based on numerous morphological characters were not retrieved as monophyletic using *cox1* sequences, even after improving the existing taxonomy based on morphological re-investigation in several cases. The presence of well-characterized species, both molecularly and morphologically, with very low divergences also confounded the use of common thresholds for our datasets, no matter whether they were defined *a priori* (clustering) or deduced from the data (GMYC).

When we compared regional subsampling of one or more areas with phylogenetic subsampling, we observed similar good average performances with respect to species richness estimation and taxonomic accuracy (Table 1). However, where clades were densely sampled at the species level, lineage-idiosyncratic data structure led to higher standard deviations for the *agreement* and *accuracy* than in the regional subsets. Thus, the analysis of single clades might suffer from high error rates due to the presence of genetically very similar or non-monophyletic sister species.

## Supporting Information

Figure S1 Sequence divergence distribution. Distribution of DNA distances among all individuals in the dataset for (blue) largest intraspecific and (orange and green) smallest congeneric, interspecific distances; (orange) raw dataset and (green) taxonomically cleaned dataset (e.g., paraphyletic species removed).
(1.31 MB TIF)

Table S1 Jackknife and bootstrap support for species, the number of species, the number of paraphyletic species in selected clades and the percentage of species with jackknife and/or bootstrap support above 50% in each clade. Jacknife values from TNT analyses (500 replicates), and bootstrap values from 250 replicates ran in GARLI.
(2.73 MB TIF)
