# Supplementary figures and images for: Mitochondrial Cox1 Sequence Data Reliably Uncover Patterns of Insect Diversity But Suffer from High Lineage-Idiosyncratic Error Rates
Source: PLoS One. 2010 Dec 28;5(12):e14448. doi: 10.1371/journal.pone.0014448 (PMC3010977; doi:10.1371/journal.pone.0014448)

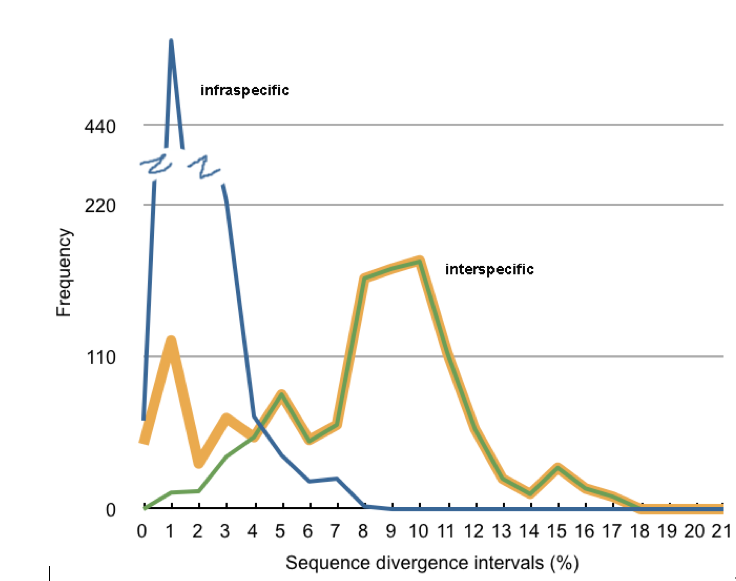

Supplement: Figure S1 — Sequence divergence distribution. Distribution of DNA distances among all individuals in the dataset for (blue) largest intraspecific and (orange and green) smallest congeneric, interspecific distances; (orange) raw dataset and (green) taxonomically cleaned dataset (e.g., paraphyletic species removed). (1.31 MB TIF) [file pone.0014448.s001.tif]

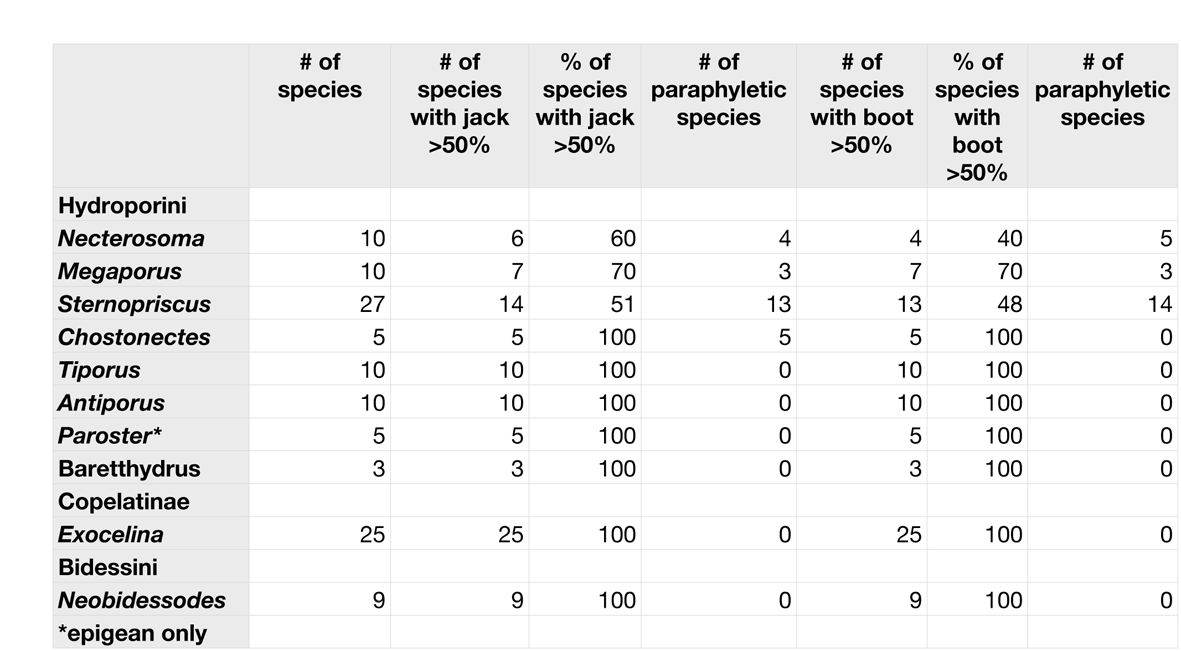

Supplement: Table S1 — Jackknife and bootstrap support for species, the number of species, the number of paraphyletic species in selected clades and the percentage of species with jackknife and/or bootstrap support above 50% in each clade. Jacknife values from TNT analyses (500 replicates), and bootstrap values from 250 replicates ran in GARLI. (2.73 MB TIF) [file pone.0014448.s002.tif]
